# Incidence of gastrointestinal stromal tumor in Chinese urban population: A national population‐based study

**DOI:** 10.1002/cam4.3644

**Published:** 2020-12-15

**Authors:** Lu Xu, Yanpeng Ma, Shengfeng Wang, Jingnan Feng, Lili Liu, Jinxi Wang, Guozhen Liu, Dianrong Xiu, Wei Fu, Siyan Zhan, Tao Sun, Pei Gao

**Affiliations:** ^1^ Department of Epidemiology and Biostatistics School of Public Health Peking University Beijing China; ^2^ Department of General Surgery Peking University Third Hospital Beijing China; ^3^ Shanghai Songsheng Business Consulting Co. Ltd Beijing China; ^4^ Peking University Health Information Technology Co. Ltd Beijing China; ^5^ Research Center of Clinical Epidemiology Peking University Third Hospital Beijing China; ^6^ Center for Intelligent Public Health Institute for Artificial Intelligence Peking University Beijing China

**Keywords:** China, epidemiology, gastrointestinal stromal tumor, incidence

## Abstract

**Background:**

Information on incidence of gastrointestinal stromal tumor (GIST), the most common type of mesenchymal tumor in gastrointestinal tract, was limited in China. This study aimed to estimate the incidence of GIST in urban population from mainland China in 2016.

**Methods:**

Urban Employee Basic Medical Insurance (UEBMI) and Urban Residence Basic Medical Insurance (URBMI) in China were used. The denominator of incidence was the total person‐years of insured individuals in 2016 in the database, covering approximately 0.43 billion individuals. The numerator was the number of incident GIST cases in 2016.

**Results:**

The crude incidence in 2016 was 0.40 per 100,000 person‐years (95% CI, 0.06–1.03). Male incidence was higher than female incidence (0.44 vs. 0.36, rate ratio: 1.22, *p* < 0.001). The mean age at diagnosis was 55.20 years (SD = 14.26) and the incidence among those aged 50 years or older was 2.63 times (0.84 vs. 0.32, *p* < 0.001) higher than those aged under 50. The highest incidence was observed in East China (2.29, 95% CI: 0.46–5.54).

**Conclusions:**

The incidence of GIST in mainland China was lower than Europe, North America and Korea. The mean age at diagnosis of GIST in China was younger than that of Europe and Canada. This study provides useful information to further research, policy formulating and management of GIST.

## Graphical abstract

This is the first study using national data in mainland China, covering 0.43 billion population, to estimate the incidence of gastrointestinal stromal tumor. The incidence in 2016 was 0.40 per 100,000 person‐years, with the age trend of incidence becoming obviously steep after 50 years old and male incidence higher than female incidence.

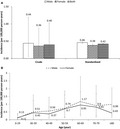

## 1 INTRODUCTION

Gastrointestinal stromal tumor (GIST) is the most common type of mesenchymal tumor in gastrointestinal tract, accounting for 5% of all sarcomas and 82% of all gastrointestinal mesenchymal tumors.^1^ GIST can occur throughout the digestive tract, mainly at the stomach (approximately 55%), followed by the small intestine (approximately 30%), colorectum (approximately 10%), and the esophagus (approximately 3%).^2^ Surgical resection is the main treatment for operable GIST^3^; however, up to 40% of GIST patients experience recurrence and metastasis after complete resection. Only approximately 70% of patients can reach 5‐year survival after operations.^4^ Meanwhile, patients with unresectable tumors and recurrent tumors are suggested to use tyrosine kinase inhibitors, resulting in a substantial economic burden on patients with the cost of more than $100,000 per patient per year.^5^

The incidences of GIST fluctuated among different countries. The incidences ranged from 0.68 to 0.70 per 100,000 person‐years in North America (i.e., Canada^6^ and USA^7^, ^8^, ^9^, ^10^), while most European countries^11^, ^12^, ^13^, ^14^, ^15^, ^16^, ^17^, ^18^, ^19^ have slightly higher incidences with a range from 1.00 to 1.45 per 100,000 person‐years.^11^, ^12^, ^13^, ^14^, ^15^, ^16^, ^17^, ^18^, ^19^ In Asia, Korea was reported to have an even higher incidence of 1.60 per 100,000 person‐years.^20^ The studies regarding incidence in China were pretty limited and inconsistent. The existing four studies were conducted only in a single city or province (i.e., Shanxi,^21^ Taiwan,^22^, ^23^ Hong Kong,^24^ and Shanghai^25^), and the incidence range was quite wide, ranging from 0.40 to 2.11 per 100,000 person‐years.

Therefore, this nationwide population‐based study was conducted to estimate the GIST incidence and analyze its patterns across sex, age, and geographical region among urban population in mainland China, using a nationally representative data from 2013 to 2016.

## 2 MATERIALS AND METHODS

### 2.1 National medical insurance database

Urban Employee Basic Medical Insurance (UEBMI) and Urban Residence Basic Medical Insurance (URBMI) are two main medical insurance schemes for urban population in China. UEBMI is for working and retired employees including employers and employees working in government agencies and institutions, state‐owned enterprises, private businesses, social organizations, as well as other private entities. URBMI is for unemployed urban residents including children, students, the elderly, etc. Each city updated the data in UEBMI and URBMI on a monthly basis. Until 2016, UEBMI and URBMI had covered over 95% of urban population in China.^26^, ^27^ The reimbursement records of the insured population will be recorded in the database, no matter the proportion they paid for the medical service. UEBMI and URBMI contain sociodemographic characteristics (nationality, sex, birth date, location, etc.), diagnostic information (disease names, disease codes, etc.), and medical expenditures of patients. This study is registered with the Chinese Clinical Trial Registry (ChiCTR), number ChiCTR1800018217.

### 2.2 Study population

A national population‐based study was carried out using the data from 23 provinces in UEBMI and URBMI between January 1st, 2013 and December 31st, 2016. Eight provinces were not included because of absence or abnormality of crucial information such as diagnostic information (Beijing, Shanghai, Sichuan, Ningxia, Hebei), only involving one insurance type (Tianjin), and reporting policy exemptions (Fujian and Tibet). All claim records were anonymous to protect patients’ privacy. The study protocol was approved by the ethical review committee of the Peking University Health Science Center (IRB. No.: IRB00001052‐18012), and the informed consent requirement was waived.

### 2.3 Case identification

Health condition at each hospital admission was identified based on the diagnostic information in the database including the diagnostic text and International Classification of Diseases (ICD) code. Natural language processing was applied to standardize the information of diagnosis with a dictionary of potential GIST defined by prestigious gastroenterologists. Potential patients with GIST were selected by ICD 10 (i.e., M8936) and Chinese medical terms of GIST. Diagnostic information of each potential patient with GIST was then reviewed by two prestigious gastroenterologists independently to identify actual target patients. All Chinese gastroenterologists made the diagnosis of GIST based on the Chinese consensus guidelines for the diagnosis and management of GIST (2013 edition)^28^ during the study period.

### 2.4 Statistical analysis

For calculation of incidence, it is necessary to ascertain the disease onset appropriately, which requires the whole disease history of an individual. However, in some settings such as using insurance data, there is no information about the disease history of an individual before the start time of the data, which results in left truncation. To handle this problem, a period before the target time point (i.e., wash‐out period) can be applied to judge the incident cases, which means only individuals free of target disease during the wash‐out period are considered as incident cases.^29^, ^30^ In this study, we used the 2013–2015 data for wash‐out to exclude the prevalent patients with GIST in 2016, since patients would visit the hospital at least once within 5 years after the diagnosis.^28^ Thus, we only calculate the incidence of GIST in 2016.

We calculated the national or regional incidence of GIST in 2016 using a two‐stage approach. In the first stage, the incidence in each province was calculated using the following method: the denominator (N) was the total person‐years of insured individuals in each province in both UEBMI and URBMI in 2016. The numerator (M) was the number of incident patients with GIST estimated from the denominator population in each province, taking the issue of missing diagnostic information into account. To be specific, the number of enrolled individuals in each province was divided into three groups: individuals with no claim records (N_1_), individuals with complete diagnostic information in their claim records (N_2_), and individuals with claim records but with missing diagnostic information (N_3_). The incident patients with GIST (M_2_) that we actually observed were from N_2_. However, several incident patients with GIST (M_3_) existed in N_3_. Since the missing diagnosis in the insurance data was mainly due to the administrative issues at prefecture‐level cities, we assumed that the probability of having GIST was not related to the missing status of diagnostic information. Thus, we considered M as (N_2_+N_3_) M_2_ /N_2_. In the second stage, we estimated the national or regional incidence by pooling the incidence of each province using the random effects meta‐analysis. To stabilize the variance of province‐specific incidence, the Freeman–Tukey double arcsine transformation was used.

Age‐adjusted incidence was estimated based on the 2010 Chinese national census data, the Revised European Standard Population (RESP) 2013, the 2010 US population and the Segi World Standard population, respectively. Subgroup analyses were conducted by sex, age, and geographical region (East, North, Northeast, Northwest, Central, South, and Southwest). We also use sensitivity analyses to assess the robustness of the primary results by only considering the observed patients with GIST to estimate the lower bound of the incidence (since we cannot rule out the possibility that there were some target patients in those with missing diagnostic information), and by excluding the top 10% of provinces with missing diagnostic information to test the robustness of the estimated rates (since different missing rates in different provinces may affect the imputed results). The unit for the incidence was per 100,000 person‐years. The 95% confidence intervals (CIs) of incidence was calculated based on the Poisson distribution. Student's t‐test for continuous variables and the chi‐squared test for categorical variables were used. All statistical analyses were done by Stata version 15.0, and a two‐sided *p* < 0.05 was considered as statistically significant.

## 3 RESULTS

In 2016, there were approximately 0.43 billion individuals in the database (Table 1). A total of 1,227 incident patients (male: 685; female: 542) with GIST were observed in the database (Table 1). The mean age at diagnosis of the observed patients was 55.20 (standard deviation, SD = 14.26) years.

**TABLE 1:** Characteristics of the incident patients with gastrointestinal stromal tumor and the entire population from urban areas of 23 provinces in China in 2016.

Characteristics		Incident patients	Entire population (million)
Total number		1,227	425.83
Sex, n (%)	Male	685 (55.83)	221.86 (52.10)
	Female	542 (44.17)	203.97 (47.90)
Age groups, n (%)	0–29	64 (5.22)	169.20 (39.73)
	30–39	128 (10.43)	66.30 (15.57)
	40–49	202 (16.46)	70.04 (16.45)
	50–59	315 (25.67)	53.06 (12.46)
	60–69	320 (26.08)	37.89 (8.90)
	70–79	157 (12.80)	19.00 (4.46)
	≥80	41 (3.34)	10.35 (2.43)
Area, n (%)	East	656 (53.46)	171.35 (40.24)
	North	2 (0.16)	18.92 (4.44)
	Northeast	10 (0.81)	42.87 (10.07)
	Northwest	11 (0.90)	21.60 (5.07)
	Central	41 (3.34)	51.26 (12.04)
	South	49 (4.00)	72.33 (16.99)
	Southwest	458 (37.33)	47.50 (11.15)

Note

East area contains Jiangsu, Zhejiang, Anhui, Jiangxi, and Shandong (five provinces). North area contains Shanxi, Inner Mongolia (two provinces). Northeast contains Liaoning, Jilin, Heilongjiang (three provinces). Northwest contains Shaanxi, Qinghai, Gansu, and Xinjiang (four provinces). Central contains Henan, Hubei, Hunan (three provinces). South contains, Guangdong, Guangxi, and Hainan (three provinces). Southwest contains Chongqing, Guizhou, and Yunnan (three provinces).

### 3.1 Incidence

**FIGURE 1:**
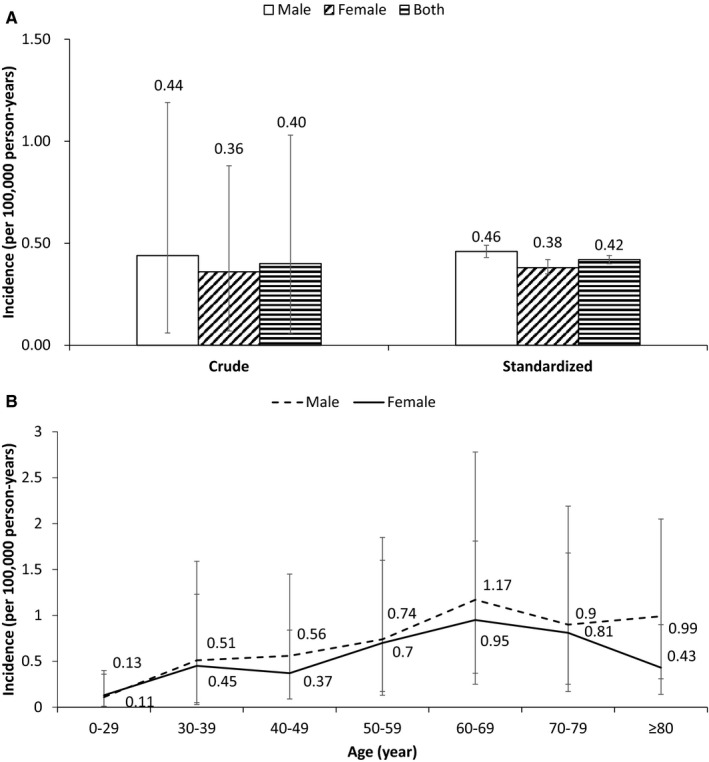
Incidence of gastrointestinal stromal tumor in urban China in 2016. Note: the standardized incidence is based on 2010 Chinese census data.

National incidence of GIST in 2016 was 0.40 per 100,000 person‐years (95% CI: 0.06–1.03) (Figure 1). Based on the Chinese census data in 2016, there were 0.79 billion urban population, therefore, in 2016, approximately 3,160 incident patients with GIST in China. Male incidence (0.44, 95% CI: 0.06–1.19) was higher than female incidence (0.36, 95% CI: 0.07–0.88), with a rate ratio of 1.22 (*p* < 0.001). The incidence peaked at 60–69 years old (1.06, 95% CI: 0.48–1.88) (Figure 1). The incidence of those aged 50 years or older was significantly higher than those aged under 50 (rate ratio: 2.63, *p* < 0.001). There were regional variations in incidence of GIST in 2016, with the highest in East China (2.29, 95% CI: 0.46–5.54) (Table 2).

**TABLE 2:** Crude incidence of gastrointestinal stromal tumor in urban population from 23 provinces in China in 2016 (unit: /100,000 person‐years).

Subgroup	Total	Male	Female
Age group			
0–29	0.12 (0.03–0.28)	0.11 (0.01–0.36)	0.13 (0.01–0.40)
30–39	0.48 (0.12–1.07)	0.51 (0.03–1.59)	0.45 (0.05–1.23)
40–49	0.46 (0.17–0.90)	0.56 (0.09–1.45)	0.37 (0.09–0.84)
50–59	0.72 (0.28–1.37)	0.74 (0.13–1.85)	0.70 (0.17–1.60)
60–69	1.06 (0.48–1.88)	1.17 (0.25–2.78)	0.95 (0.37–1.81)
70–79	0.85 (0.36–1.56)	0.90 (0.17–2.19)	0.81 (0.25–1.68)
≥80	0.68 (0.32–1.18)	0.99 (0.31–2.05)	0.43 (0.14–0.90)
Area			
East	2.29 (0.46–5.54)	2.78 (0.03–10.10)	1.85 (0.07–6.05)
North	0.02 (0.00–0.06)	0.01 (0.00–0.03)	0.04 (0.00–0.19)
Northeast	0.05 (0.00–0.13)	0.06 (0.00–0.23)	0.04 (0.00–0.19)
Northwest	0.10 (0.02–0.27)	0.11 (0.00–0.42)	0.10 (0.00–0.37)
Central	0.21 (0.02–0.86)	0.22 (0.01–1.03)	0.20 (0.00–0.79)
South	0.13 (0.10–0.16)	0.15 (0.11–0.19)	0.11 (0.07–0.15)
Southwest	0.63 (0.14–1.50)	0.54 (0.00–2.17)	0.73 (0.00–2.68)

### 3.2 Standardized incidence

The age‐adjusted national incidence based on 2010 Chinese census data was 0.42 per 100,000 person‐years (95% CI: 0.40–0.44), with 0.46 (95% CI: 0.43–0.49) and 0.38 (95% CI: 0.35–0.42) for males and females, respectively. The standardized rates based on the US, European populations and the Segi World Standard population can be seen in Table 3 and the standardized rates were slightly higher than the corresponding crude rates, except the standardized rates based on the Segi World Standard population.

**TABLE 3:** Age‐adjusted incidence of gastrointestinal stromal tumor in urban population from 23 provinces in China in 2016 (unit: /100,000 person‐years).

Reference population	Standardized incidence (95% CI)		
	Total	Male	Female
2010 Chinese census data	0.42 (0.40–0.44)	0.46 (0.43–0.49)	0.38 (0.35–0.42)
RESP 2013 census data	0.50 (0.48–0.52)	0.55 (0.52–0.59)	0.45 (0.42–0.49)
2010 US census data	0.45 (0.42–0.47)	0.49 (0.46–0.52)	0.41 (0.38–0.44)
Segi World Standard population	0.35 (0.43–0.48)	0.38 (0.47–0.53)	0.33 (0.38–0.44)

Abbreviation: RESP, Revised European Standard Population.

### 3.3 Sensitivity analysis

Lower bound of the national incidence was 0.16 per 100,000 person‐years (95% CI: 0.05–0.34) by only considering the observed patients, which are thought to be underestimated (Table 4). The national incidence calculated by excluding the top 10% of provinces with missing diagnostic information (i.e., Shandong and Xinjiang) was 0.42 (95% CI: 0.04–1.22).

**TABLE 4:** Sensitivity analyses for crude incidence of gastrointestinal stromal tumor in urban population from 23 provinces in China in 2016 (unit: /100,000 person‐years).

	Using only observed cases^a^	Excluding the top 10% of provinces with missing diagnostic information^b^
Male	0.18 (0.05–0.38)	0.47 (0.03–1.42)
Female	0.16 (0.05–0.33)	0.38 (0.05–1.03)
Total	0.16 (0.05–0.34)	0.42 (0.04–1.22)

^a^ Known to be an underestimation of incidences.

^b^ Shandong and Xinjiang were excluded.

## 4 DISCUSSION

Our study is the first to evaluate the incidence of GIST in mainland China. The crude incidence was 0.40 per 100,000 person‐years in urban China in 2016, with the incidence increasing with age. In addition, male predominance and regional variations in incidence of GIST were observed in China.

The crude incidence of GIST in our study was slightly lower than Europe countries^11^, ^12^, ^13^, ^14^, ^15^, ^16^, ^17^, ^18^, ^19^, ^31^ and North America,^6^, ^7^, ^8^, ^9^, ^10^ and significantly lower than Korea,^20^ but within the range of previous reported regional incidences in China.^21^, ^25^, ^32^ In our main analyses, the number of target patients with missing diagnostic information was imputed by assuming that the probability of having gastrointestinal stromal tumor was not related to the missing status of diagnostic information. We also did two sensitivity analyses: only considering the observed patients with GIST to estimate the lower bound of the incidence; and excluding the top 10% of provinces with missing diagnostic information to test the robustness of the estimated rates. The result from the second sensitivity analysis was similar with the main result, suggesting that the level of missing rate had little influence on the imputed results. All three results were lower than the incidences in other countries which ranged from 0.65 per 100,000 person‐years to 2.20 per 100,000 person‐years.^6^, ^7^, ^8^, ^9^, ^10^, ^11^, ^12^, ^13^, ^14^, ^15^, ^16^, ^17^, ^18^, ^19^, ^20^, ^23^, ^31^, ^33^ A couple of underlying reasons might explain those disparities. First, the lesions for many asymptomatic patients with GIST could be found accidentally in endoscopic screening or bariatric surgery. The nationwide health check‐up program for gastric cancer since 1999 in Korea^34^ and the popularity of bariatric surgery since 1954 in Europe and North America^35^ might contribute to the relatively higher incidences. In contrast, the cancer screening program in urban China, initiated in 2012, has not covered the whole population and bariatric surgery is still nascent with a surgery quantity of only 4200 cases per year in China.^36^, ^37^ Second, differences in economic and medical level among different countries might also result in this disparity. The relatively limited immunohistochemistry equipment for diagnosis, insufficient understanding of the pathobiology are still existing in the underdeveloped regions in China, which will inevitably lead to the misdiagnosis in those areas.^38^, ^39^ This potential reason had been reported in previous studies.^21^, ^25^ Third, we could not exclude the possibility that the relatively higher incidences in Europe, North America, and Korea might also result from the higher prevalence of the risk factors for GIST in these countries, such as aging population, western diet, tobacco use, and alcohol intake.^40^, ^41^, ^42^ As these factors are also relatively prevalent in East China,^43^, ^44^ our study found a higher incidence for GIST in this region. And the standardized rates based on the US, European populations were slightly higher than the corresponding crude rates, while the standardized rates based on the Segi World Standard population was lower. That may be associated with population aging in developed countries.

Consistent with previous studies in either other countries^6^, ^7^, ^8^, ^9^, ^19^ or China,^21^, ^25^ our study reported an upward trend in the age‐specific incidence for GIST in mainland China, which became obviously steep after 50 years old. Furthermore, in accordance with previous studies from USA,^10^ Canada,^6^ and Korea,^45^ our results found people over the age of 50 were 2.63 times more likely to develop GIST than those aged under 50. It is widely accepted that the accumulation of age‐related mutations can be observed in cancerous tumors due to general problems in DNA repair mechanisms.^46^ And previous studies^46^, ^47^ found that c‐KIT mutation in somatic cells, which is the most important determinants in the tumorigenesis of GIST,^48^ was significantly higher in patients above 50 years old compared with younger patients. In addition, the mean age at diagnosis was 55.20 years in our study, consistent with the previous reports in China^21^, ^25^ and Korea,^20^, ^45^ but approximately one decade younger than Europe^11^, ^12^, ^13^, ^14^, ^15^, ^16^, ^17^, ^18^, ^19^, ^31^, ^33^ and North America.^6^, ^7^, ^8^, ^9^, ^10^ Given the fact that age at onset is proportional to age at diagnosis, the shorter life expectancy in China compared with Europe and North America^49^ may partly explain the younger age at diagnosis.^50^ As for Korea, although it has a similar life expectancy with Europe and North America, more asymptomatic younger patients, which were identified by the nationwide health check‐up program for gastric cancer, may decrease the mean age. Besides, a distinct molecular subgroup of wild‐type GIST, characterized by succinate dehydrogenase subunit deficiency, usually occurs in children and young adults.^51^ However, the proportion of this subgroup were not reported in previous studies and it was also not mentioned in the database used in our study. Further investigations focused on this subgroup of GIST are needed.

In our results, a significant male predominance for incidence of GIST in mainland China was observed, similar with USA,^7^, ^10^ Korea,^20^ and Taiwan.^22^ Although four studies from Spain^16^, ^17^ and China^21^, ^25^ indicated no gender differences, the relatively small sample size in these four studies^16^, ^17^, ^21^, ^25^ may lead to the unmanifested gender variation. Previous studies^31^, ^52^ found highly malignant tumors were even more than twice as common in males than in females, and further research^53^, ^54^ showed that this phenomenon may be due to the extreme down‐regulation of chromosome Y gene expression and Escape from X‐Inactivation Tumor Suppressor genes mutations. Furthermore, drinking and smoking, which are more common in males, are risk factors for malignant tumors.^40^, ^41^ Thus, the male predominance might truly exist in GIST like in other malignant tumors.

To our knowledge, this is the first nationwide population‐based study to analyze the incidence patterns across sex, age group, and geographical region of GIST in mainland China. Our large national sample size ensured relatively stable and convincing results. Nevertheless, this study also has several limitations. First, the wash‐out period used to exclude the prevalent patients with GIST might be not sufficient. However, this period was defined following the clinical practice, since patients are required to be followed up at least once annually within 5 years after the diagnosis of GIST according to *Chinese consensus guidelines for diagnosis and management of gastrointestinal stromal tumor*.^28^ Second, the diverse missing proportions of diagnosis‐related variables might affect the estimation. However, several sensitivity analyses were conducted to explore the potential influences on the estimations. In particular, the lower bound of the incidence was presented using only observed cases of GIST which could facilitate the interpretation of the findings. Third, compared with registry studies, studies based on claim data may be less effective for case ascertainment, but claim data can ensure a large sample for studies on rare conditions.^55^ Forth, for the patients with GIST who visited private institutions, if they did not show their insurance card at their visits to these institutions, they would not be identified as patients with GIST in claim data, which might cause underestimation of the incidence of GIST. But the proportion of this population should be small. Finally, although this study covered more than 95% urban population, certain people such as military soldiers were not included in the study due to the different types of medical insurance programs, but after standardized based on 2010 Chinese census data, there was negligible variations in the incidences.

In conclusion, we conducted a nationwide study using claims data to investigate the incidence of GIST in mainland China in 2016. The incidence of GIST in mainland China was lower than Europe, North America, and Korea, but it varied among different regions in China, with the highest in East China. The incidence became obviously steep after 50 years old and the mean age of diagnosis of GIST in mainland China was younger than that in Europe and Canada. Males had a higher incidence than females in this study. These findings lay the foundation for the further clinical and basic studies of GIST, and are helpful for formulating GIST related policies.

## CONFLICT OF INTEREST

The authors declare no conflict of interest.

## AUTHOR’S CONTRIBUTIONS

Shengfeng Wang, Siyan Zhan, Tao Sun and Pei Gao: Conceptualization; Jinxi Wang and Guozhen Liu: Data curation; Lu Xu: Formal analysis; Siyan Zhan and Wei Fu: Funding acquisition; Lu Xu, Siyan Zhan, Tao Sun, Dianrong Xiu, Wei Fu and Shengfeng Wang: Investigation; Siyan Zhan, Lu Xu, Pei Gao and Shengfeng Wang: Methodology; Siyan Zhan and Shengfeng Wang: Project administration; Siyan Zhan: Resources; Lu Xu: Software; Siyan Zhan, Tao Sun and Shengfeng Wang: Supervision; Lu Xu and Pei Gao: Validation; Lu Xu, Jingnan Feng and Lili Liu: Visualization; Lu Xu, Yanpeng Ma and Shengfeng Wang: Roles/Writing ‐ original draft; All authors: Writing ‐ review & editing.

## FUNDING INFORMATION

This work was supported by the National Natural Science Foundation of China (grant numbers 91646107, 81973146, 81672361).

## Data Availability

The data that support the findings of this study are available on request from the corresponding author. The data are not publicly available due to privacy or ethical restrictions.
